# A Compressed Sensing Approach for Multiple Obstacle Localisation Using Sonar Sensors in Air

**DOI:** 10.3390/s20195511

**Published:** 2020-09-26

**Authors:** Eduardo Tondin Ferreira Dias, Hugo Vieira Neto, Fábio Kurt Schneider

**Affiliations:** Graduate Program in Electrical and Computer Engineering (CPGEI), Federal University of Technology-Paraná (UTFPR), Curitiba-PR 80230-901, Brazil; hvieir@utfpr.edu.br (H.V.N.); fabioks@utfpr.edu.br (F.K.S.)

**Keywords:** compressed sensing, sonar imaging, environment mapping

## Abstract

Methods for autonomous navigation systems using sonars in air traditionally use the time-of-flight technique for obstacle detection and environment mapping. However, this technique suffers from constructive and destructive interference of ultrasonic reflections from multiple obstacles in the environment, requiring several acquisitions for proper mapping. This paper presents a novel approach for obstacle detection and localisation using inverse problems and compressed sensing concepts. Experiments were conducted with multiple obstacles present in a controlled environment using a hardware platform with four transducers, which was specially designed for sending, receiving and acquiring raw ultrasonic signals. A comparison between the performance of compressed sensing using Orthogonal Matching Pursuit and two traditional image reconstruction methods was conducted. The reconstructed 2D images representing the cross-section of the sensed environment were quantitatively assessed, showing promising results for robotic mapping tasks using compressed sensing.

## 1. Introduction

Navigation systems such as autonomous or remotely operated vehicles in general, including mobile robots, traditionally use lidar, radar or sonar sensors for object localisation and environment mapping. Lidar sensing provides high angular resolution when compared to radar and sonar sensing, but has difficulties in dealing with dusty, smoky or foggy environments, highly polished surfaces or optically transparent materials, such as glass [1,2]. On the other hand, radar and sonar sensing do not suffer interference from environmental visual conditions, but these modalities have difficulties in dealing with the occurrence of multiple signal reflections in the environment [3,4]. Nevertheless, sonar sensing is a well-known and widespread technique for environment mapping due to the cost-effectiveness and relative easiness of implementation of the necessary hardware, when compared to radar and lidar sensing [1,5].

The conventional detection method in sonar-based autonomous navigation uses the time-of-flight (TOF) concept, which consists of measuring the travel time between the signal burst sent to the environment and the detection of its reflections (echoes) from the existing obstacles [1,6]. This technique, when performed with a single ultrasonic sensor, results in small angular coverage due to the typical main lobe sensitivity characteristics of ultrasound transducers, as well as undesired artefacts due to side lobe characteristics. As a solution, a large array of sonars is normally used for TOF obstacle detection. However, adding more sonars to the detection system also causes difficulties in discriminating true obstacle reflections due to crosstalk, multiple reflections in the environment, and constructive/destructive interference [3,4].

Traditional approaches to solve these problems focus on minimising crosstalk and noise using probabilistic methods [7] such as occupancy grids [8,9], Kalman filtering [7,10] or geometric-based feature detection [11,12]. A novel approach presented in [13] used the inverse problem approach for the generation of 2D images-an occupancy grip map-representing the cross-section of the sensed environment, based on the premise that raw ultrasonic reflection signals contain relevant environmental information. The method uses a small array of off-the-shelf sonar sensors to generate maps without the need of multiple and successive sensor readings. Experiments were performed using traditional inverse problem algorithms with compelling results for a single obstacle present in a real environment.

In related work, [14] uses a compressed sensing method for image reconstruction. The technique uses a single sonar sensor for acquiring the obstacle reflection in the environment and generating maps. Experiments were performed with three obstacles distributed sparsely in a simulated environment, and results demonstrated the possibility of using Orthogonal Matching Pursuit (OMP) algorithms for robotic mapping. That article also discusses the problem of symmetry in the reconstructed maps due to the use of a single sonar and interference between the obstacles. Further research following this approach was presented in [15], in which the main goal was to recover the sonar signal using compressed sensing methods in a real environment in low-power and low-memory systems. A controlled environment with up to two obstacles far from a single sonar sensor was used, and experiments were performed: single obstacle at different distances and different angles, and with two obstacles present in the environment. Two different algorithms were used, Basis Pursuit and Matching Pursuit, with a sub-sampled basis for the reconstruction signals.

In [16], a discussion about the use of a compressed sensing approach for wideband sonar imaging is presented. The approach consists in a modified compressed sensing algorithm-using a $\ell_{1}$-norm minimisation in the first pass of the algorithm-for image reconstruction using simulated data for Doppler frequency shifts and delays. An extension of the research is presented in [17], in which there is an analysis of the effects of off-grid obstacles using traditional compressed sensing for the reconstruction.

The present paper addresses obstacle detection and localisation for robotic mapping applications using compressed sensing concepts and sonar sensors operating in air. A specific hardware platform was designed and implemented to send, receive and simultaneously acquire raw reflections from four ultrasonic transducers. Experiments were conducted in a real environment containing one, two and three standard cylindrical obstacles (2.5 cm diameter, 25 cm height) placed in different configurations. The OMP algorithm [18] was implemented to reconstruct the 2D image corresponding to the cross-section of the environment. Additionally, two traditional image reconstruction methods, based on the Moore–Penrose Inverse [19] and Tikhonov Regularisation [20], were also implemented and used to compare image reconstruction performances.

All algorithms were implemented using both real- and complex-valued (Hilbert transform [21]) models and three methods were used to assess image reconstruction accuracy: (1) exact image reconstruction score, (2) number of detected obstacles and (3) the Euclidean distance between the coordinates of pixels in the reconstructed image that represent detected obstacles and the coordinates of pixels in the ground-truth image that represent actual obstacles.

The remainder of the paper is structured as follows: Section 2 describes the theoretical foundations of reconstruction methods based on the inverse problem approach, as well as the compressed sensing method. The experimental setup is described in Section 3, while results are presented and discussed in Section 4. Section 5 presents conclusions and future work.

## 2. Inverse Problems

Environment mapping using sonar sensors and the inverse problem approach can be mathematically modelled using Equation (1):

(1) g=Hf+e,

in which $f\in R^{n}$ is the vector version of the 2D ground-truth image representing the amplitude map of reflections from objects, $g\in R^{m}$ is the sample vector read from the sensor array, the $H\in R^{m\times n}$ matrix represents the discrete acquisition model and $e\in R^{m}$ is the acquisition noise vector [22].

In sonar imaging, the point spread function (PSF) defines the response of ultrasonic reflections received from a particular obstacle to a given position in a region of interest (ROI) [23]. The *H* matrix is modelled by placing these responses-*m* samples of the signal acquired along time for each of the *n* ROI mapped locations-in each of its columns. For systems with more than one transducer, the PSF is formed by concatenation of the reflection signals received from each transducer. This process is based on medical ultrasound imaging research and is detailed in [24,25,26,27].

### 2.1. Least Squares Method

A simple technique to solve the linear system in Equation (1) is by inverting the *H* matrix, such that $\hat{f}=H^{-1}g$. However, this solution is valid only for ideal systems, in which the *H* matrix has an inverse $H^{-1}$ and the acquisition noise *e* is known. Reconstruction methods aim to find a solution to the resulting inverse problem by estimating a vector *f* that satisfies the linear system. Traditional methods are based on minimising a cost function, such as the least squares (LS) approximation [28]:

(2) $$\hat{f}=\underset{f}{\arg\min}||g-{Hf||}_{2}^{2},$$

in which $||g-{Hf||}_{2}$ is the $\ell_{2}$ norm of the residual error vector.

A well-known solution is based on a deterministic model that estimates the vector $\hat{f}$ using a closed expression. Equation (3) describes this inversion process:

(3) $$\hat{f}={\left(H^{T}H\right)}^{-1}H^{T}g,$$

in which $H^{T}$ is the transpose matrix of *H* and ${\left(H^{T}H\right)}^{-1}H^{T}$ is the Moore–Penrose inverse matrix of *H* [29].

However, due to ill-conditioning of *H*, this deterministic model becomes unstable, and noise amplified, causing artefacts in the reconstructed image [30]. To deal with this problem, linear regularisation methods, such as Total Variation [31] and Tikhonov Regularisation [25], are used. These methods add a regularisation parameter to the system to minimise the resulting noise amplification. In this work, the Tikhonov Regularisation method was used, which is mathematically defined in Equation (4):

(4) $$\hat{f}=\underset{f}{\arg\min}||g-{Hf||}_{2}^{2}+{\lambda ||\Gamma f||}_{2}^{2},$$

in which Γ is the Tikhonov matrix and λ is the regularisation parameter. The closed-form solution is given in Equation (5):

(5) $$\hat{f}={\left(H^{T}H+\lambda \Gamma^{T}\Gamma \right)}^{-1}H^{T}g.$$

In some cases, such as in ultrasonic systems, the identity matrix (I) may be used as Γ. A new Tikhonov mathematical formulation is then defined by:

(6) $$\hat{f}=\underset{f}{\arg\min}||g-{Hf||}_{2}+{\lambda ||f||}_{2}^{2},$$

in which λ controls the sensitivity between the residual error norm ($||g-{Hf||}_{2}^{2}$) and the regularisation term (${\left||f|\right|}_{2}$) [32,33]. Therefore, it is essential to choose a well fitted λ for an accurate solution. Different methods can be used to estimate this parameter, such as Generalised Cross-Validation [34] and the L-Curve [32]. In this work, the L-Curve method was used.

### 2.2. Orthogonal Matching Pursuit

Compressed sensing methods rely on the sparse characteristics of the signal of interest and small inaccurate measurements in the acquired signal [35,36]. This approach is based on the premise that real systems present important sparse information.

Compressed sensing provides several approaches to solve the reconstruction problem [37,38], such as convex optimisations, greedy algorithms and Bayesian methods. Greedy algorithms have shown success in medical imaging [26] and studies with radar and sonar imaging systems [14,15,17].

The mathematical formulation of the method is given by:

(7) $$\begin{array}{cccc} \; & \underset{f}{\mathrm{minimise}}\; & \; & {\left||f|\right|}_{1}\\ \; & \mathrm{subject}\;\mathrm{to}\; & \; & ||g-{Hf||}_{2}\leq \epsilon , \end{array}$$

in which ϵ is a constraint.

The greedy algorithm used in this work to solve Equation (7) was the Orthogonal Matching Pursuit (OMP) [18,39]. OMP performs, in each iteration, inner products between the residual error and the columns of the *H* matrix in order to select the column *k* that corresponds to the maximum correlation [17,38], as shown in Equation (8):

(8) $$k=\underset{i}{\arg\max}\left\{|H^{T}r_{i}|\right\}.$$

The selected column *k* (also referred to as atom) is added to a support matrix *S* and the signal is reconstructed using the pseudo-inverse solution, as shown in Equation (3). Then, the residual error is updated using Equation (9) until the stop criterion is satisfied:

(9) $$r=g-H_{S}f_{S}^{*}.$$

Two stop criteria were used in this work: the number of iterations and the level of novelty [40]. The first criterion can be used when the sparsity of the system is previously known. However, in autonomous mobile robots, this information can not be known beforehand, and it is desired that the algorithm will be able to stop automatically. With that in mind, a new criterion was defined based on the level of novelty, defined as the difference between the previous residual error norm and the current residual error norm. The algorithm stops when the level of novelty reaches a predefined threshold.

The pseudocode in Algorithm 1 gives a brief description of the OMP implementation used in this work.
**Algorithm 1:** Orthogonal Matching Pursuit
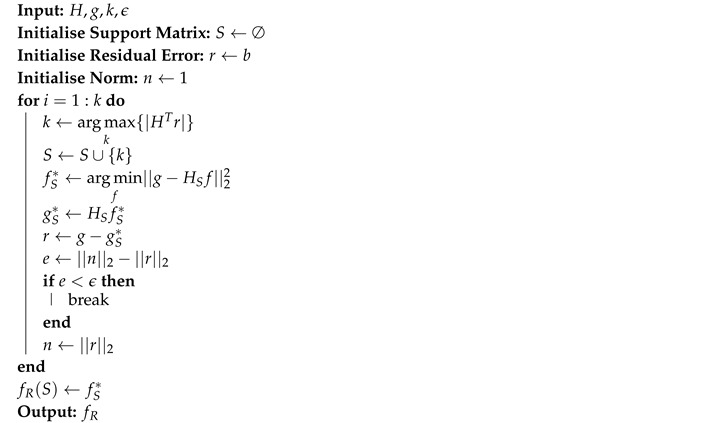


## 3. Experimental Setup

### 3.1. Acquisition System

**Figure 1:**
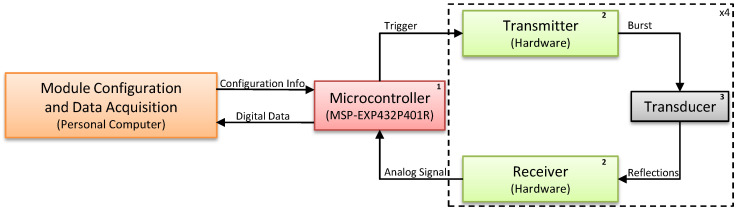
Multiple obstacle detection system block diagram.

A hardware platform for multiple obstacle detection was developed for the acquisition of raw ultrasonic signals. A variety of transducer configurations can be set, such as simultaneous or delayed activation of each transducer, the number of cycles in the ultrasonic signal burst, and the number of transducers set to receive the reflected burst. For instance, the hardware platform makes it possible to send an ultrasonic burst using two transducers and to acquire the raw signal of its reflection from the environment simultaneously using up to four transducers. The platform consists of hardware for transmitting and receiving ultrasonic waves and a control application running in a host PC for parameter setup and data storage. Figure 1 shows the block diagram of the designed platform. The set of parameters defined in the control application are sent to an MSP432 microcontroller (1), which sends triggering signals to the transmitter module (2), exciting the desired transducers (3) and sending a 50 kHz ultrasound wave to the environment. Reflections received by the transducers are processed by the receiver module and acquired by the microcontroller, which then sends the data to the host PC for storage and processing.

**Figure 2:**
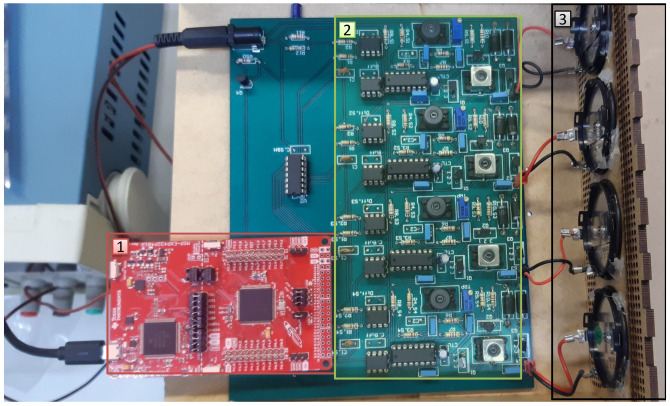
The hardware module designed for ultrasonic wave transmission and reception.

The hardware has an array of four 50 kHz electrostatic transducers, circuits for transducer excitation and circuits for amplification and sampling of received signals. The Texas Instruments MSP-EXP432P401R Launchpad^TM^ development kit (http://www.ti.com/tool/MSP-EXP432P401R) was used to trigger the transducers, acquire the received signals after amplification and sampling, and send the acquired data to the PC for storage and processing. Figure 2 shows the hardware that was developed, which consists of: (1) a Texas Instruments MSP432 microcontroller; (2) the transmission and reception hardware; and (3) four electrostatic transducers. The numbered rectangles in Figure 2 correspond to the numbered blocks in Figure 1.

In this work, the hardware was configured to send 16 burst cycles from individual transducers and to acquire the reflected signal in all of the four available transducers. The signals received in each transducer were sampled at 200 kHz and composed signal vectors of 1024 samples, corresponding to a period of 5.12 ms, which allows the detection of objects located as far as 165 cm from the transducers. In this configuration, the period of the ultrasound echo corresponds to 64 samples in the acquired signal vector.

### 3.2. Acquisition Setup and Data Collection

The controlled experimental environment defined in this work consists of a surface area of 30 cm ×125 cm with a square grid of possible positions for obstacles spaced by 2.5 cm. However, the ROI for image reconstruction in the scope of this work was limited to a grid of 5×5 (10 cm ×10 cm) possible positions for obstacles, in order to simplify assessment and visualisation of experimental results. In more realistic application scenarios, the ROI can be expanded to the limits in which ultrasound reflections can be detected by the hardware.

**Figure 3:**
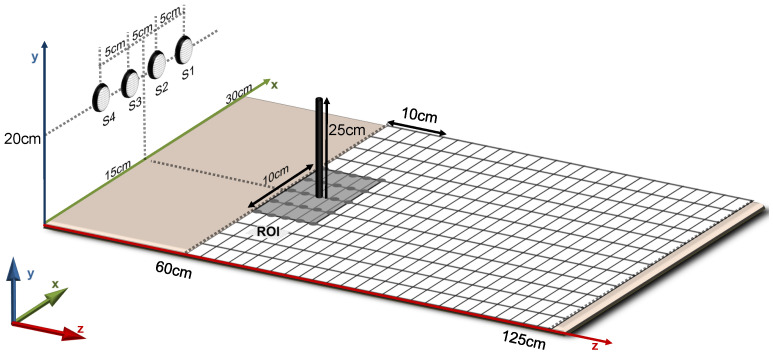
Controlled experimental environment sketch.

Figure 3 presents a sketch of the controlled experimental environment. Four transducers were symmetrically positioned in front of the grid at a distance of 60 cm, with their centres spaced by 5 cm from each other, at 20 cm height from the surface of the grid. Cylindrical obstacles with 25 cm height and 2.5 cm diameter were used in the experiments reported in this work.

The linear system identification, i.e., construction of the *H* matrix, was performed by triggering each transducer individually and acquiring the corresponding reflections from all of the four available transducers simultaneously. The four acquired signals corresponding to each obstacle location in the grid were concatenated into single column vectors and appended side by side as columns of the *H* matrix. The process was performed for all 25 ROI positions and repeated for each transducer that was triggered, resulting in an *H* matrix with 4×4×1024=16,384 rows and 25 columns. Since the desired 2D image is reordered in vector form (*f*) and each pixel represents a location in the ROI, the number of columns (*n*) in *H* corresponds to the number of reconstructed image pixels in *f*.

**Figure 4:**
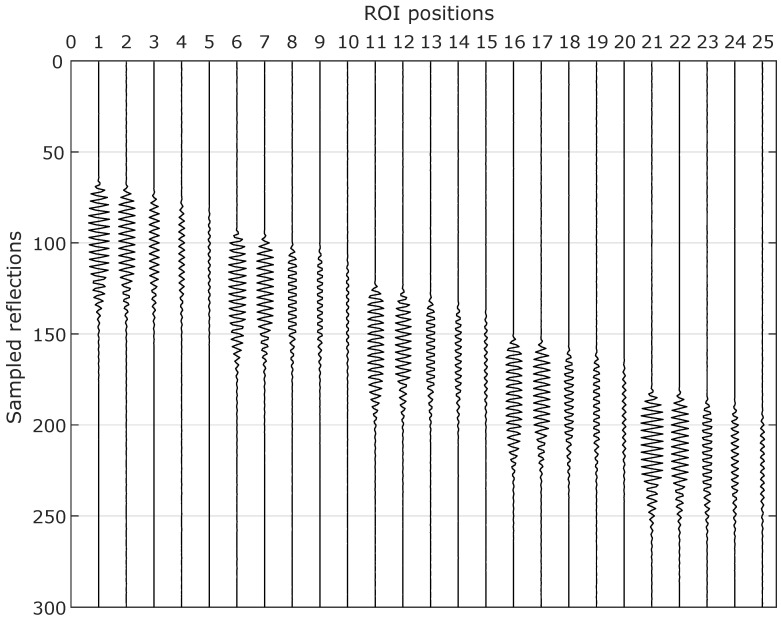
Linear system identification: the *H* matrix is constructed with the PSF for a single obstacle placed in each ROI position.

The first 300 rows of the *H* matrix are depicted in graphic format in Figure 4 and correspond to the signals acquired from transducer S1. The columns correspond to a single obstacle placed in different ROI positions (starting from the closest ROI location to S1, see Figure 3).

It can be noticed in Figure 4 that not only burst detection delays increase (as in the TOF approach), but also signal amplitudes decrease as the distance between the obstacle and transducer S1 increases. Similar patterns occur for transducers S2, S3 and S4 in lower rows of the *H* matrix (not shown in Figure 4).

**Figure 5:**
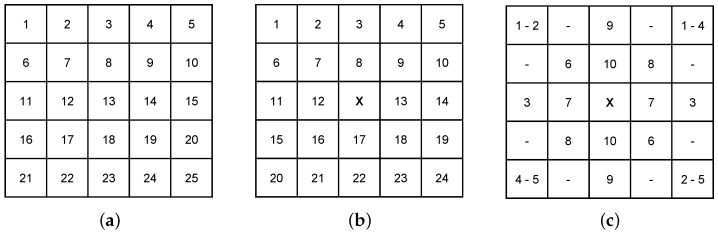
Positioning of obstacles for the reconstruction experiments: (**a**) a single obstacle was positioned in one of the possible ROI positions. (**b**) One obstacle was fixed at the ROI centre (X) and another was placed in one of the remaining possible positions. (**c**) Three obstacles were used, with one fixed at the ROI central position and the other two placed in the positions indicated by numbers 1 to 10.

Image reconstructions were performed with one, two or three obstacles placed in distinct configurations within the ROI. Figure 5a illustrates the configuration used when a single obstacle was positioned in the environment—all the available positions were used in the assessments. For the reconstructions containing two obstacles, as shown in Figure 5b, while an obstacle was fixed at the central position of the ROI, another one was positioned in the remaining spots. The experiments with three obstacles were made according to Figure 5c. The image reconstructions were performed for a total of 25+24+10=59 obstacles configurations.

## 4. Experimental Results

Experiments were made to assess the capabilities of the image reconstruction algorithms aiming at environment mapping. Three algorithms were implemented: Pseudo-Inverse (PINV), Tikhonov (TIKH) and Orthogonal Matching Pursuit (OMP). A Hilbert Transform was performed in the raw signal for enhancing algorithms results. The algorithms were executed using real-valued data (*-R) and complex-valued data (*-C) obtained from the Hilbert Transform (the * represents the algorithm used—e.g., the Pseudo-Inverse algorithm using the real-valued model is referred to as PINV-R).

**Figure 6:**
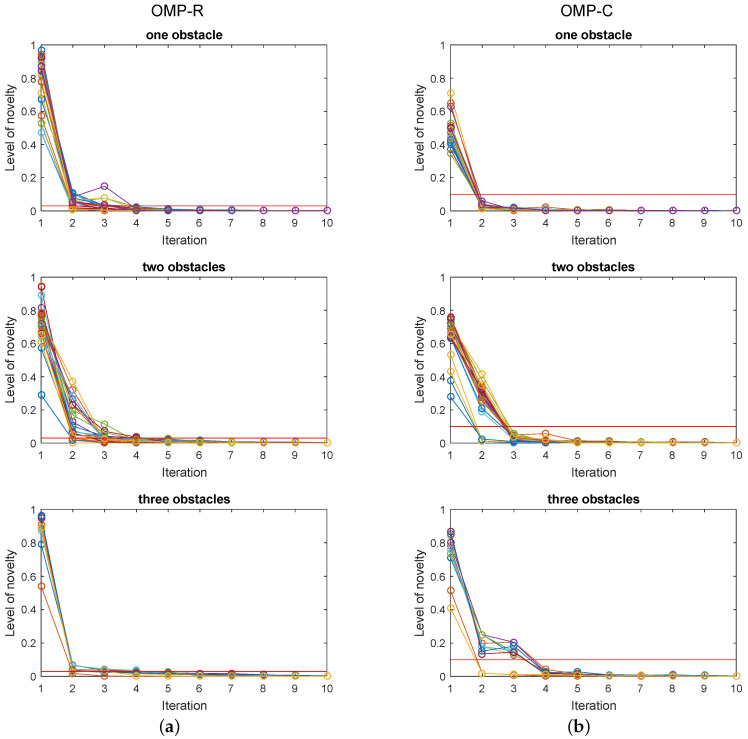
Level of novelty in 10 iterations for all OMP reconstructions: (**a**) corresponds to real-valued model results, and (**b**) to complex-valued model results. The red line corresponds to the chosen novelty levels, 0.03 for OMP-R and 0.1 for OMP-C.

The regularisation parameter value was 0.5626 and 0.3106 for TIKH-R and TIKH-C algorithms, respectively. These values were defined using the L-Curve method [41]-the same value was obtained for the experiments with one, two or three obstacles in the environment. The level of novelty chosen for the stop criterion was 0.03 and 0.1 for OMP-R and OMP-C algorithms, respectively. Both values were defined by graphical analysis of novelty. Figure 6 presents the level of novelty for all OMP reconstruction in 10 iterations. Figure 6a shows the real-valued model results for the three experiments and Figure 6b presents the complex-valued model. The red lines in the graphs indicate the chosen novelty levels.

The number of iterations used as stop criterion was set for a maximum of 10 iterations, but none of the OMP algorithms has ever reached this number of iterations.

### 4.1. Image Reconstruction

**Figure 7:**
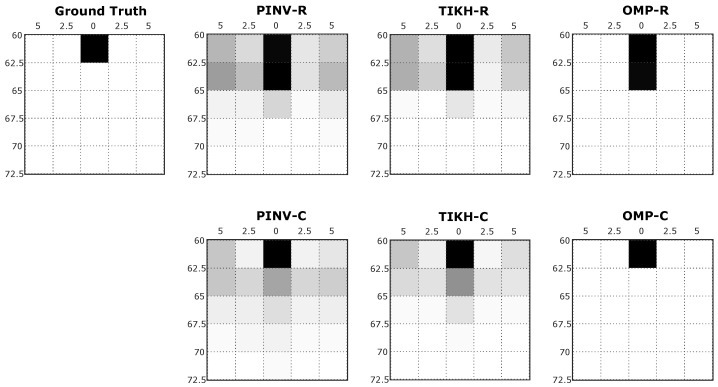
Examples of reconstructed images with a single obstacle in the environment.

The grey scale images resulting from the reconstruction process represent the cross-section of the sensed environment. While white pixels represent empty spaces in the grid, black pixels indicate high probability of a real obstacle being present in the corresponding grid coordinates. Figure 7 shows examples of reconstructed images generated in the first experiment, with just one obstacle placed at 60 cm from the transducers at position 3 in Figure 5a.

For the specific position shown in Figure 7, traditional reconstruction algorithms, Pseudo-Inverse and Tikhonov presented artefacts in the resulting environment mapping, using either real-valued or complex-valued models. This occurs mainly because both algorithms were not able to deal efficiently with the influence of noise during signal acquisition or even during the identification of the linear system (*H* matrix). The compressed sensing OMP algorithm, on the other hand, minimised the effects of noise and the presence of artefacts. All algorithms using the real-valued model reconstructed the environment with an additional non existing obstacle when compared to the ground truth. The OMP algorithm was able to reconstruct an image in complete agreement with the expected ground truth when using the complex-valued model.

**Figure 8:**
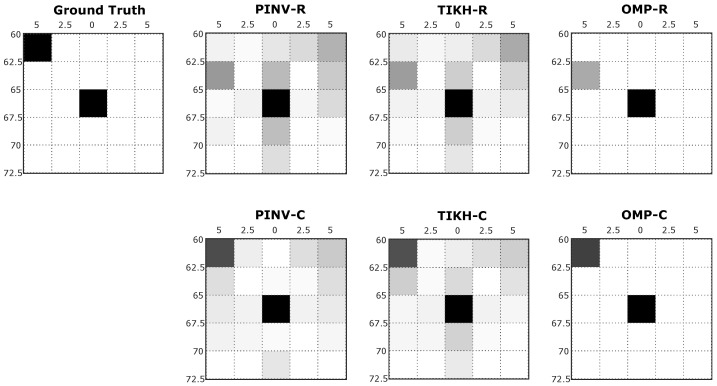
Examples of reconstructed images with two obstacles in the environment.

Examples of reconstructed images with two obstacles in the environment (obstacles placed at positions 1 and X in Figure 5b) are shown in Figure 8, in which one can notice a similar effect to what occurred in the single obstacle experiment. PINV-R and TIKH-R (real-valued model) presented artefacts and misplaced one of the obstacles in the grid. PINV-C and TIKH-C (complex-valued model) correctly positioned all the obstacles in the grid, but still with the presence of artefacts. The OMP-R eliminated the artefacts but misplaced one of the objects. The OMP-C algorithm was able to reconstruct the image corresponding to the environment correctly.

**Figure 9:**
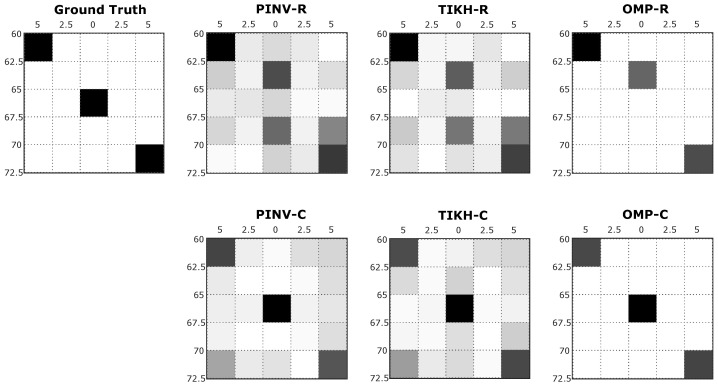
Examples of reconstructed images with three obstacles in the environment.

Figure 9 shows image reconstructions for the experiment with three obstacles in the environment. The PINV-R and TIKH-R presented artefacts and failed to reconstruct the obstacle at the central position (see Figure 5c). The PINV-C and TIKH-C algorithms were able to reconstruct all the obstacles, but some minor artefacts were also observed. OMP-R reconstructed all the three obstacles, but a displacement in the obstacle located at the centre was noticed. Finally, OMP-C correctly reconstructed all three objects in the environment.

Both traditional methods, PINV and TIKH, use all columns of *H* during the inversion process. This process leads to the occurrence of artefacts in the reconstructed images due to disturbances in the model identification process. On the other hand, the compressed sensing OMP method uses only the columns of *H* (atoms) with the highest correlation with the acquired input signal, drastically reducing artefacts in the reconstructed images. For a fair comparison analysis of results, focusing on the localisation of the obstacles, the reconstructed images by the PINV and TIKH algorithms were thresholded using the values shown in Table 1. The maximum pixel intensity values for thresholding for each algorithm were obtained by Receiver Operating Characteristics (ROC) curves [42].

**Figure 10:**
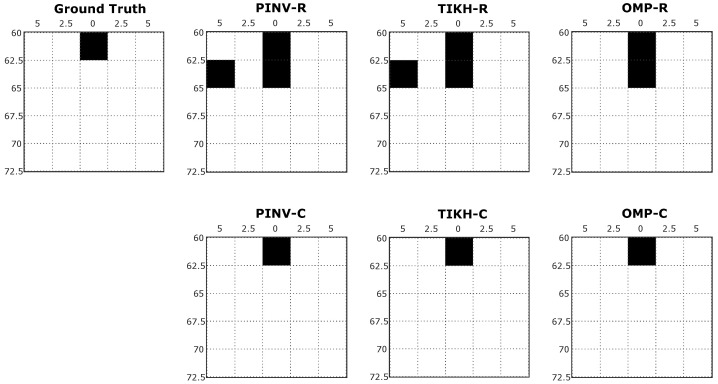
Examples of reconstructed images with a single obstacle in the environment after thresholding.

Figure 10 shows the reconstructed images after thresholding for the single obstacle experiment. In this example, as expected, the presence of noisy responses was minimised in reconstructions using the PINV and TIKH algorithms. Although the reconstructed images were thresholded, the algorithms that used the real-valued model still reconstructed more obstacles than the actual number of obstacles present in the environment. On the other hand, all the algorithms that used the complex-valued model were able to get the correct result after thresholding.

**Figure 11:**
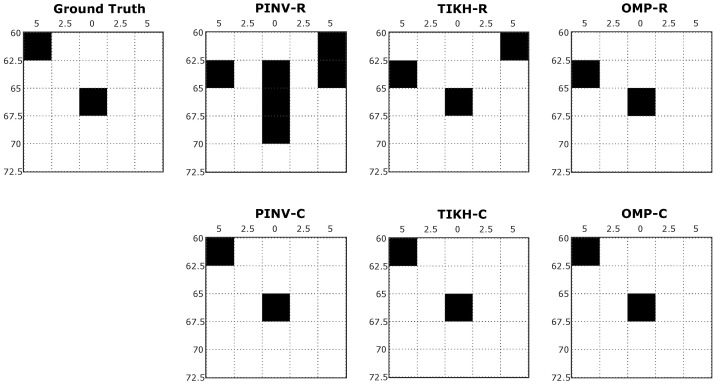
Examples of reconstructed image with two obstacles in the environment after thresholding.

The reconstructed images after thresholding, for the experiments with two obstacles in the environment, are shown in Figure 11. The algorithms using the complex-valued model presented the best results, which precisely correspond to the ground-truth, while the ones that used the real-valued model still presented reconstruction errors.

**Figure 12:**
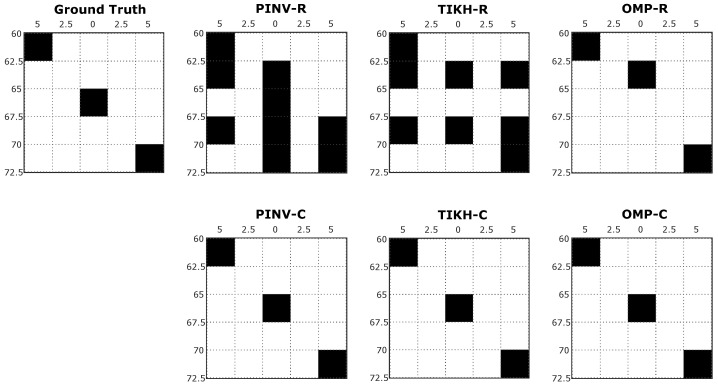
Examples of reconstructed images with three obstacles in the environment after thresholding.

The experiment with three obstacles in the environment presented similar results to the other experiments, as shown in Figure 12. A significant increase in false positives were observed when using the real-valued model for PINV and TIKH. Once again, all algorithms reconstructed the environment correctly when using the complex-valued model.

### 4.2. Performance Assessments

Three different performance analyses were conducted. The first assessment aims at quantifying the similarity between the reconstructed images and the actual environment. The second is intended to quantify reconstruction errors that were preliminarily noticed—for that, obstacles in the reconstructed images were characterised as true positives or false positives. Finally, the Euclidean distance between the coordinates of reconstructed obstacles and the coordinates of actual obstacles in the environment was computed in the third assessment. A non-parametric test was also performed to determine the statistically significant differences between the results.

#### 4.2.1. Reconstruction Score

The first assessment consists of a similarity measurement between the reconstructed image and the expected ground truth. A comparison between the thresholded version of the reconstructed image and the ground-truth yields a binary result for this assessment statistic, valued one in case of exact image reconstruction or zero otherwise. All individual comparison results are then added up to compute a single reconstruction score for each algorithm.

**Figure 13:**
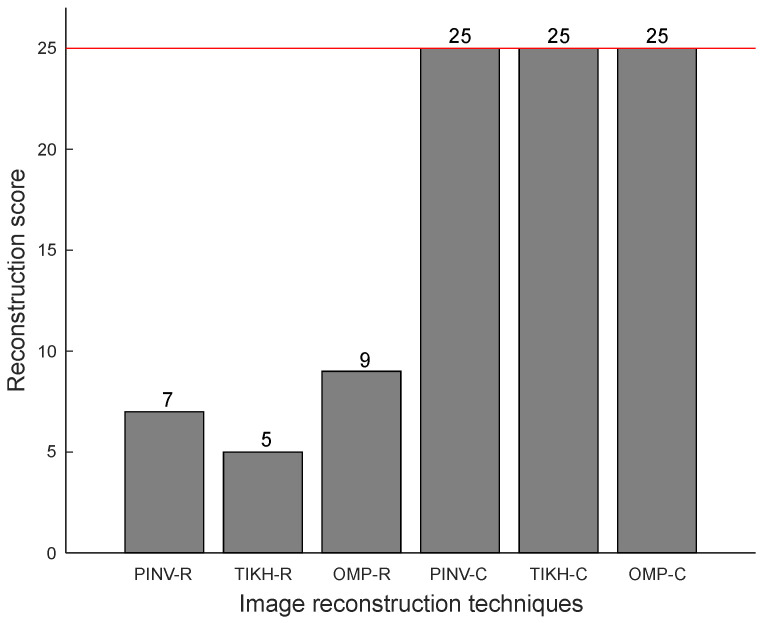
Reconstruction scores with a single obstacle in the environment.

For the experiments with a single obstacle in the environment, a perfect reconstruction score would be 25, as there are 25 possible locations for a single object in the grid (see Figure 5). Figure 13 shows the reconstruction scores obtained for the experiments with a single obstacle, in which the red line indicates the maximum possible value for the reconstruction score.

All images reconstructed by the algorithms using the complex-valued model are identical to their respective ground-truth images and correspond to accurate representations of the environment (perfect reconstruction scores) in all cases. On the other hand, reconstructions using the real-valued model fail to be perfect in more than two thirds of cases. The results in Figure 13 illustrate the positive impact of the Hilbert Transform (complex-valued model) in the accuracy of reconstructions with a single object in the environment.

**Figure 14:**
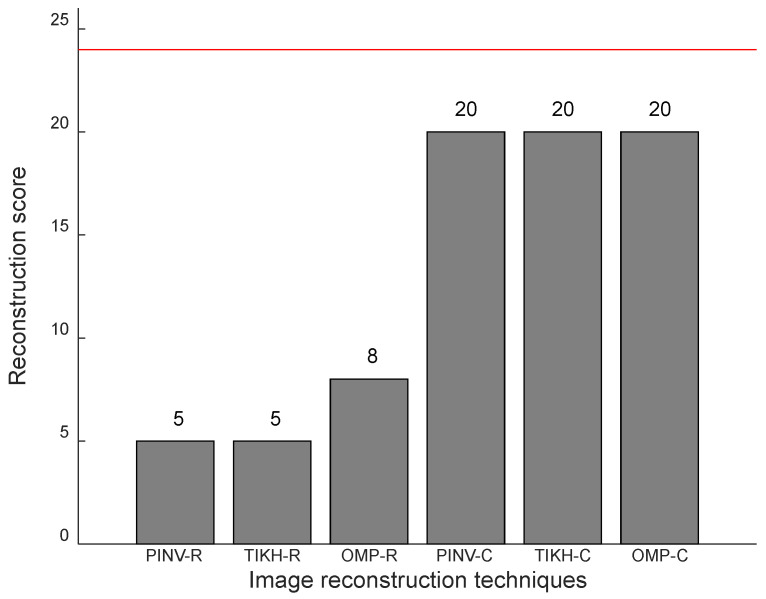
Reconstruction scores with two obstacles in the environment.

Figure 14 presents the results for the experiments with two obstacles in the environment, in which the maximum possible reconstruction score is 24. Once again, the algorithms using the complex-valued model yielded the best results, although four obstacle configurations were not reconstructed correctly. The algorithms using the real-valued model failed at least in two thirds of cases.

**Figure 15:**
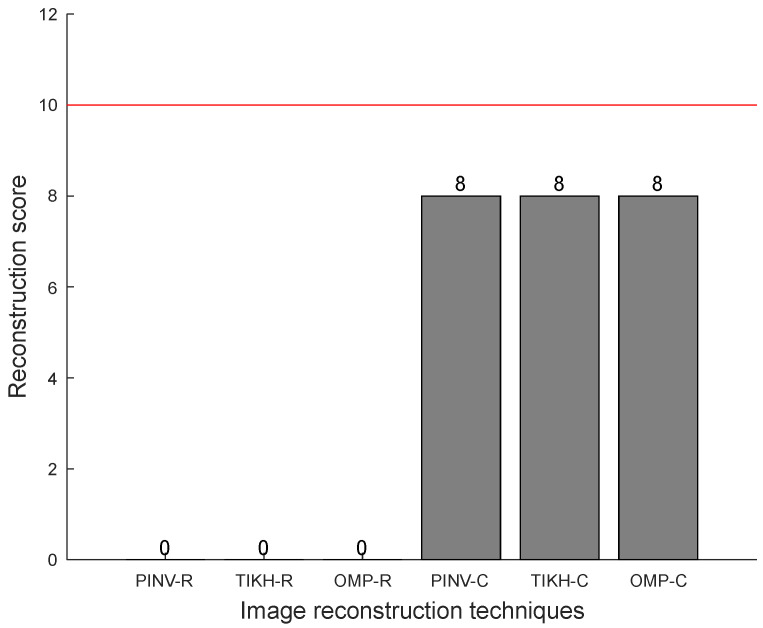
Reconstruction scores with three obstacles in the environment.

The reconstruction scores for three obstacles in the environment can be seen in Figure 15, in which one can notice that all algorithms using the complex-valued model yielded the best results—a score of 8 out of a maximum possible value of 10, indicated by the red line. Algorithms that used the real-valued model failed completely in the task of accurately reconstructing three obstacles in the environment.

The first assessment indicated that the use of the complex-valued model results in a more accurate image reconstruction when compared to the use of the real-valued model. A rate of 100% exact reconstructions was observed for the cases with only one obstacle in the environment. The presence of more than one obstacle made image reconstruction more difficult, as expected. However, PINV-C, TIKH-C and OMP-C algorithms were able to accurately reconstruct 82.35% of cases.

#### 4.2.2. Number of True Positives and False Positives

The second assessment analysed the number of true positives and false positives regarding the obstacles detected by the algorithms. A true positive is the occurrence of a black pixel indicating the presence of an obstacle at the correct location of the reconstructed image. On the other hand, the occurrence of a black pixel indicating the presence of an obstacle at an incorrect location of the reconstructed image is a false positive. Table 2 shows the results obtained by all algorithms for the number of detected obstacles, assuming a total of 25, 24 and 10 reconstructions for the experiments with one, two and three obstacles in the environment, respectively.

The reconstructed images using the real-valued model present false positives due to the presence of noisy artefacts. The PINV-R and TIKH-R algorithms have shown a large percentage of false positives in the experiments with three obstacles, revealing weaknesses of these algorithms when the number of objects in the environment increases.

The algorithms that used the complex-valued model correctly positioned the obstacle in all 25 reconstructed images in the single obstacle experiments, as already verified in the first assessment. In the experiments with two and three obstacles, no false positives were observed, although the results of true positives were 91.7% and 86.7% for two and three obstacles, respectively. The absence of false positives demonstrates that the algorithms have missed obstacles in four of the reconstructions with two obstacles and two of the reconstructions with three obstacles present, which correspond to the errors observed in the reconstruction scores (first assessment).

**Figure 16:**
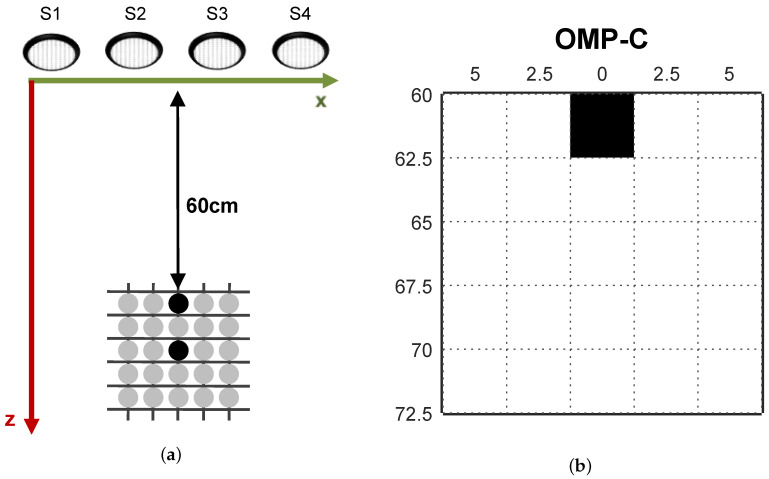
Image reconstruction error: an example of a missing object when two obstacles are in the same longitudinal axis. (**a**) presents the position of the obstacles with reference to the transducers, and (**b**) presents the resulting OMP-C reconstruction.

Further analysis of the reconstructed images revealed that missing obstacle errors occur when the obstacles in the environment are in the same longitudinal axis (one behind the other from the point of view of the transducers). In this particular spatial configuration of the objects in the environment, the algorithms were able to reconstruct only the closest obstacle to the transducers. Figure 16a shows an example of spatial configuration of two objects in which the furthest is missing from the reconstruction. Figure 16b presents the OMP-C reconstruction with the missing central obstacle. The same behaviour for this particular spatial configuration is observed in experiments with two or three obstacles for all algorithms using the complex-valued model.

The kind of reconstruction error shown in Figure 16 happens due to the low level of novelty between the signals acquired by the transducers. The closest obstacle reflects most of the emitted energy, while reflections from further obstacles in the same longitudinal axis are not recognised by the algorithms. This phenomenon can be thought as the closest object “casting a shadow” over the objects behind.

Regarding false positives, which appear in reconstructions with the algorithms using the real-valued model, they are due to the presence of noisy artefacts and justify the low score shown by the algorithms in the first performance assessment (reconstruction score). The algorithms using the real-valued model are not able to deal with noise adequately, especially when there is an increase in the number of obstacles in the environment. The results obtained by the algorithms using the complex-valued model, on the other hand, had no noise artefacts and consequently had no false positives either. However, obstacles were missed when they shared the same longitudinal axis in the environment.

#### 4.2.3. Euclidean Distance

The third assessment was an analysis of the Euclidean distance between pixel coordinates of reconstructed obstacles and the actual coordinates of obstacles in the ground-truth. The pixels with the highest intensity and only the exact number of obstacles present in the environment were considered in this assessment. Each pixel in the reconstructed image corresponds to a distance of 2.5 cm in the real environment. The best result for the Euclidean distance metric is zero, indicating that the obstacles are correctly positioned with reference to the ground-truth.

**Figure 17:**
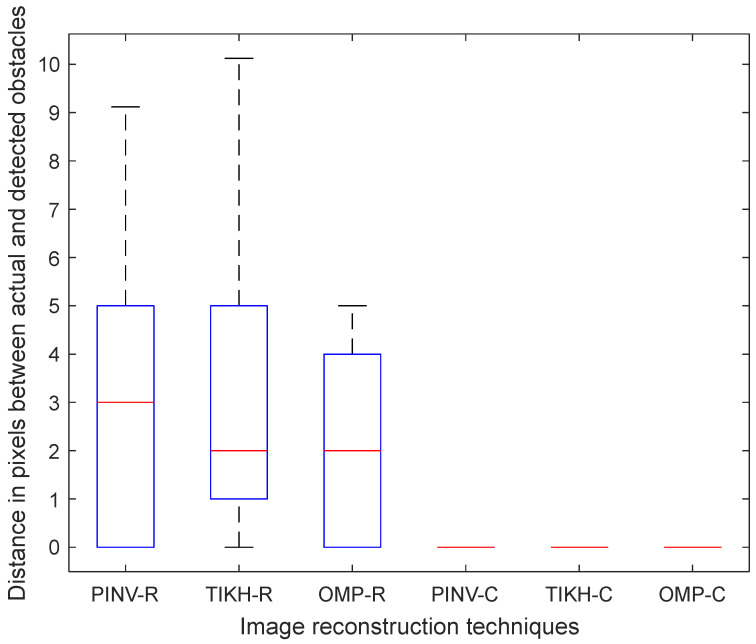
Euclidean distance in pixels between the single reconstructed obstacle coordinates and its actual coordinates in the ground-truth.

Figure 17 shows the Boxplot of the median Euclidean distance distribution for the single obstacle experiment. All obstacles were correctly positioned by the algorithms using the complex-valued model. On the other hand, the algorithms using the real-valued model misplaced the obstacle—three pixels for PINV-R and two pixels by TIKH-R and OMP-R, on average.

**Figure 18:**
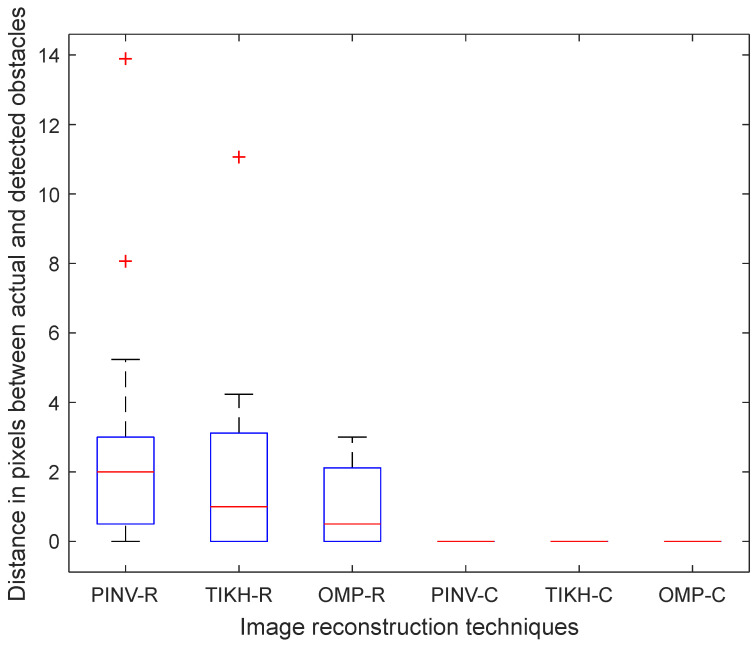
Euclidean distance in pixels between two reconstructed obstacle coordinates and their actual coordinates in the ground-truth.

The results for the experiments with two obstacles in the environment were similar to the ones for the experiments with a single obstacle, as shown in the Figure 18. The algorithms using the real-valued model still misplaced obstacles, although to a lesser extent on average. All detected obstacles were correctly positioned by the algorithms using the complex-valued model.

**Figure 19:**
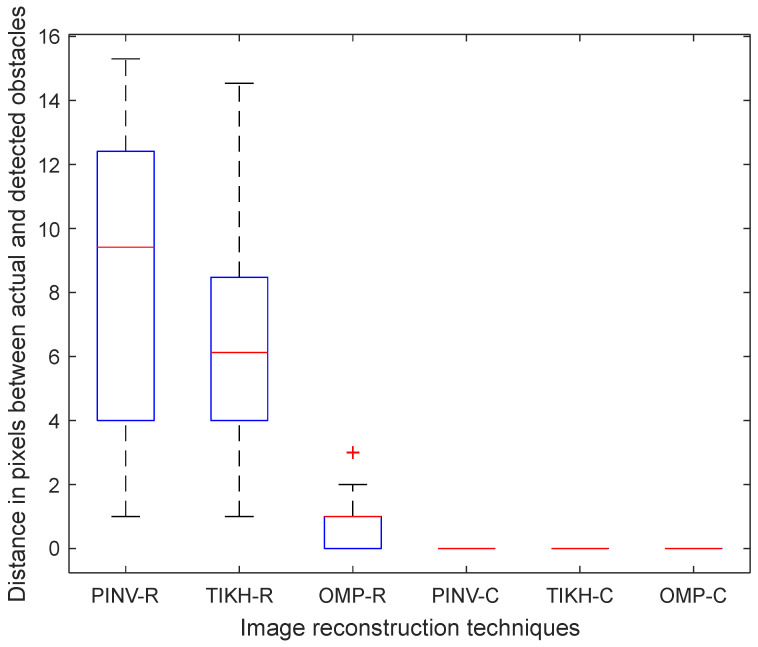
Euclidean distance in pixels between three reconstructed obstacle coordinates and their actual coordinates in the ground-truth.

Finally, the results for the experiments with three obstacles are shown in Figure 19. In this case, the Euclidean distance error increased for algorithms PINV-R and TIKH-R, on average. However, the average distance for the OMP-R algorithm was slightly better than in the experiments with two obstacles. The complex-valued model once again yielded the best results.

The non-parametric Kruskal–Wallis test was performed to determine whether there are statistically significant differences between performances regarding the Euclidean distance assessment. The statistical analysis for the experiment with a single obstacle in the environment showed no difference between the algorithms using the real-valued model, but there is statistically significant difference between results obtained using the real- and complex-valued models. The same effect was observed in experiments with two obstacles. Concerning the experiment with three obstacles, the statistical analysis of the Euclidean distance assessment revealed three statistically different performance groups: (1) PINV-R and TIKH-R had the worst performance; (2) OMP-R showed better performance than PINV-R and TIKH-R; and (3) PINV-C, TIKH-C and OMP-C yielded the best results.

#### 4.2.4. General Discussion of Results

The assessments demonstrated that the algorithms using the real-valued model, PINV-R, TIKH-R, and OMP-R are not able to deal efficiently with signal acquisition noise and consequently are not capable of reconstructing the experimental environments correctly. Noise artefacts (false positives) and misplaced obstacles are observed even after PINV and TIKH reconstructed images were thresholded. The regularisation parameter used by the TIKH-R algorithm did not improve performance, with results close to ones obtained for PINV-R, and despite the suppressed artefacts by OMP-R, the algorithm still fails in most reconstructions.

On the other hand, the algorithms using the complex-valued model consistently presented the best results in all assessments, albeit they presented a very specific problem—only the closest obstacle was detected in reconstructions of the environments with two and three obstacles present when they shared the same longitudinal axis. Although this problem influences the analyses of results, it can be addressed in robotic mapping, for example, by moving the robot position and acquiring a second environment reading. Another solution is to use time correction for reflections. However, due to the lack of prior knowledge of the environment, time correction was not used in the experiments.

## 5. Conclusions and Future Work

Autonomous systems must allow reliable navigation without colliding with obstacles. Environment mapping using sonar in the air traditionally use the time-of-flight technique to detect obstacles, but suffers from measurement errors caused by interference from multiple obstacles present in the environment. Consequently, the represented environment maps have low resolution and need multiple scans of the environment for proper mapping.

A novel approach initiated in [13] has shown promising results using inverse problem methods to reconstruct an environment with a single obstacle. The present paper develops this approach further and presents a comparison of a compressed sensing algorithm (OMP) with two traditional inverse problem algorithms, for multiple obstacle localisation using a small array of sonar sensors and without the need for multiple environment scans.

Reconstructions were performed for one, two and three obstacles in a controlled testing environment using a platform developed for acquisition of ultrasonic reflections. Three algorithms were implemented and the images reconstructed using real and complex-valued models. The experiments were analysed using three assessment methods and presented promising results for use in obstacle localisation. PINV-C, TIKH-C and OMP-C algorithms, all of them using complex-valued models, presented similar results. However, due to better treatment of sparsity by compressed sensing algorithms and the absence of post-processing, OMP-C is considered the best algorithm for obstacle localisation using sonars in air.

Future work aims to overcome the existing detection problem that arises when obstacles are positioned precisely in the same longitudinal axis, including experiments with other greedy algorithms such as Orthogonal Least Squares (OLS) [43], especially in environments densely populated with off-grid obstacles. The implementation of the reconstruction algorithms in an embedded system to be installed in autonomous mobile robots is also subject of future work. 

## Figures and Tables

**Table 1:** Maximum pixel intensity value obtained from ROC curves for thresholding the reconstructed images by PINV and TIKH algorithms.

Algorithm	One Obstacle	Two Obstacles	Three Obstacles
PINV-R	0.30	0.20	0.15
TIKH-R	0.30	0.20	0.15
PINV-C	0.70	0.45	0.50
TIKH-C	0.70	0.45	0.50

**Table 2:** Percentage of true positives and false positives regarding detected obstacles.

Algorithm	One Obstacle		Two Obstacles		Three Obstacles	
	TP	FP	TP	FP	TP	FP
PINV-R	84.0%	6.83%	81.3%	7.97%	83.3%	30.00%
TIKH-R	80.0%	7.17%	79.2%	6.34%	76.7%	24.55%
OMP-R	84.0%	4.50%	75.0%	2.90%	53.3%	4.55%
PINV-C	100%	0.00%	91.7%	0.00%	86.7%	0.00%
TIKH-C	100%	0.00%	91.7%	0.00%	86.7%	0.00%
OMP-C	100%	0.00%	91.7%	0.00%	86.7%	0.00%

## References

[1] Siegwart R., Nourbakhsh I.R., Scaramuzza D. (2011). Introduction to Autonomous Mobile Robots.

[2] Fritsche P., Kueppers S., Briese G., Wagner B. Radar and LiDAR Sensorfusion in Low Visibility Environments. Proceedings of the 13th International Conference on Informatics in Control, Automation and Robotics.

[3] Jörg K.W., Berg M. Mobile Robot Sonar Sensing with Pseudo-random Codes. Proceedings of the 1998 IEEE International Conference on Robotics and Automation.

[4] Bank D., Kämpke T. (2007). High-Resolution Ultrasonic Environment Imaging. IEEE Trans. Rob..

[5] Kapoor R., Ramasamy S., Gardi A., Bieber C., Silverberg L., Sabatini R. (2016). A Novel 3D Multilateration Sensor using Distributed Ultrasonic Beacons for Indoor Navigation. Sensors.

[6] Everett H.R. (1995). Sensors for Mobile Robots: Theory and Application.

[7] Thrun S. (2002). Exploring Artificial Intelligence in the New Millenium, Chapter Robotic Mapping: A Survey.

[8] Moravec H., Elfes A. High Resolution Maps from Wide Angle Sonar. Proceedings of the IEEE International Conference on Robotics and Automation.

[9] Elfes A. (1987). Sonar-based Real-world Mapping and Navigation. Int. J. Rob. Autom..

[10] Kalman R.E. (1960). A New Approach to Linear Filtering and Prediction Problems. J. Basic Eng..

[11] Jimenez J., Urena J., Mazo M., Hernandez A., Santiso E. Three-dimensional Discrimination between Planes, Corners and Edges using Ultrasonic Sensors. Proceedings of the 2003 IEEE Conference on Emerging Technologies and Factory Automation.

[12] Martínez M., Benet G. (2010). Wall-Corner Classification using Sonar: A New Approach Based on Geometric Features. Sensors.

[13] Dias E.T.F., Vieira Neto H. (2015). A Novel Approach to Environment Mapping using Sonar Sensors and Inverse Problems. Towards Autonomous Robotic Systems.

[14] Sanchez S.R., Andersson S.B. Using Compressive Sensing With In-air Ultrasonic Measurements for Robotic Mapping. Proceedings of the ASME 2018 Dynamic Systems and Control Conference.

[15] Pinto S., Sanchez S.R., Doran L., Ryan A., Andersson S.B. (2019). Reconstruction of Ultrasound Signals using Randomly Acquired Samples in a Sparse Environment. IFAC-PapersOnLine.

[16] Yan H., Peng S., Zhu Z., Xu J., Zhang X. Wideband Sonar Imaging via Compressed Sensing. Proceedings of the OCEANS 2014-TAIPEI.

[17] Stanković I., Ioana C., Daković M., Stanković L. Analysis of Off-grid Effects in Wideband Sonar Images using Compressive Sensing. Proceedings of the OCEANS 2018 MTS/IEEE Charleston.

[18] Tropp J.A., Gilbert A.C. (2007). Signal Recovery from Random Measurements via Orthogonal Matching Pursuit. IEEE Trans. Inf. Theory.

[19] Penrose R. (1955). A Generalized Inverse for Matrices. Math. Proc. Cambridge Philos. Soc..

[20] Tikhonov A.N., Goncharsky A.V., Stepanov V.V., Yagola A.G. (2013). Numerical Methods for the Solution of Ill-Posed Problems.

[21] Oppenheim A.V., Schafer R.W. (2009). Discrete-Time Signal Processing.

[22] Barrett H.H., Myers K.J., Rathee S. (2004). Foundations of Image Science.

[23] Jensen J.A. (1991). A Model for The Propagation and Scattering of Ultrasound in Tissue. J. Acoust. Soc..

[24] Jensen J.A. (2001). Linear Description of Ultrasound Imaging Systems: Notes for the International Summer School on Advanced Ultrasound Imaging at the Technical University of Denmark.

[25] Lingvall F., Olofsson T. (2007). On Time-domain Model-based Ultrasonic Array Imaging. IEEE Trans. Ultrason. Ferroelectr. Freq. Control.

[26] Passarin T.R., Zibetti M.W., Pipa D.R. (2018). Sparse Ultrasound Imaging via Manifold Low-Rank Approximation and Non-Convex Greedy Pursuit. Sensors.

[27] Viola F., Ellis M.A., Walker W.F. (2008). Time-domain Optimized Near-field Estimator for Ultrasound Imaging: Initial Development and Results. IEEE Trans. Med. Imaging.

[28] Björck A. (1996). Numerical Methods for Least Squares Problems.

[29] Bertero M. (1989). Linear Inverse and Ill-posed Problems. Adv. Electron. Electron Phys..

[30] Hansen P. (1998). Rank-Deficient and Discrete Ill-Posed Problems: Numerical Aspects of Linear Inversion.

[31] Rudin L.I., Osher S., Fatemi E. (1992). Nonlinear Total Variation Based Noise Removal Algorithms. Phys. D Nonlinear Phenom..

[32] Hansen P.C. (1992). Analysis of Discrete Ill-posed Problems by Means of the L-curve. SIAM Rev..

[33] Valente S., Zibetti M.W., Pipa D.R., Maia J.M., Schneider F.K. (2017). An Assessment of Iterative Reconstruction Methods for Sparse Ultrasound Imaging. Sensors.

[34] Golub G.H., Heath M., Wahba G. (1979). Generalized Cross-validation as a Method for Choosing a Good Ridge Parameter. Technometrics.

[35] Donoho D.L. (2006). Compressed Sensing. IEEE Trans. Inf. Theory.

[36] Candes E.J., Wakin M.B. (2008). An Introduction to Compressive Sampling. IEEE Signal Process Mag..

[37] Orović I., Papić V., Ioana C., Li X., Stanković S. (2016). Compressive Sensing in Signal Processing: Algorithms and Transform Domain Formulations. Math. Probl. Eng..

[38] Arjoune Y., Kaabouch N., El Ghazi H., Tamtaoui A. Compressive Sensing: Performance Comparison of Sparse Recovery Algorithms. Proceedings of the 7th IEEE Annual Computing and Communication Workshop and Conference.

[39] Tropp J.A. (2004). Greed is Good: Algorithmic Results for Sparse Approximation. IEEE Trans. Inf. Theory.

[40] Vieira Neto H., Nehmzow U. Incremental PCA: An Alternative Approach for Novelty Detection. Proceedings of the TAROS 2005: Towards Autonomous Robotic Systems.

[41] Hansen P.C. (1994). Regularization Tools: A Matlab Package for Analysis and Solution of Discrete Ill-posed Problems. Numer. Algorithms.

[42] Fawcett T. (2006). An Introduction to ROC Analysis. Pattern Recognit. Lett..

[43] Chen S., Billings S.A., Luo W. (1989). Orthogonal Least Squares Methods and Their Application to Non-linear System Identification. Int. J. Controll.

